# Prior to the Oral Therapy, What Do We Know About HCV-4 in Egypt: A Randomized Survey of Prevalence and Risks Using Data Mining Computed Analysis

**DOI:** 10.1097/01.md.0000464205.70920.83

**Published:** 2015-01-16

In the article “Prior to the Oral Therapy, What Do We Know About HCV-4 in Egypt: A Randomized Survey of Prevalence and Risks Using Data Mining Computed Analysis”^1^ which appeared in Volume 93, Issue 28 of *Medicine*, Abd Elrazek Abd Elrazek's name incorrectly appeared as Abd Elrazek M. Aly Abd Elrazek. The article has since been corrected and reposted online.
